# Ceftazidime-avibactam induced renal disorders: past and present

**DOI:** 10.3389/fphar.2024.1329307

**Published:** 2024-01-22

**Authors:** Yanrong Shi, Jichao Wu, Wei Mi, Xusheng Zhang, Xiuli Ren, Chengwu Shen, Cuicui Lu

**Affiliations:** ^1^ Department of Pharmacy, Shandong Provincial Hospital Affiliated to Shandong First Medical University, Jinan, China; ^2^ Department of Pharmacology, School of Basic Medical Sciences, Shandong University, Jinan, China

**Keywords:** ceftazidime-avibactam, acute kidney injury, clinical trials, real-world studies, pharmacokinetic/pharmacodynamic

## Abstract

With the increasing prevalence of multidrug-resistant Gram-negative bacterial pathogens worldwide, antimicrobial resistance has become a significant public health concern. Ceftazidime-avibactam (CAZ-AVI) exhibited excellent *in vitro* activity against many carbapenemase-producing pathogens, and was widely used for the treatment of various complicated infections. CAZ-AVI is well tolerated across all dosing regimens, and its associated acute kidney injury (AKI) in phase II/III clinical trials is rare. However, recent real-world studies have demonstrated that CAZ-AVI associated AKI was more frequent in real-world than in phase II and III clinical trials, particularly in patients receiving concomitant nephrotoxic agents, with critically ill patients being at a higher risk. Herein, we reviewed the safety data related to renal impairment of CAZ-AVI, and discussed its pharmacokinetic/pharmacodynamic targets and dosage adjustment in patients with impaired renal function. This review aimed to emphasize the importance for healthcare professionals to be aware of this adverse event of CAZ-AVI and provide practical insights into the dosage optimization in critically ill patients with renal dysfunction.

## 1 Introduction

Antimicrobial resistance has become a significant global public health concern, with the escalating prevalence of multidrug-resistant (MDR) Gram-negative bacterial pathogens worldwide, especially of carbapenemase-producing pathogens, including carbapenem-resistant *Enterobacteriaceae* (CRE), carbapenem-resistant *Pseudomonas aeruginosa* (CRPA), and carbapenem-resistant *Acinetobacter baumannii* (CRAB) (Zowawi et al., 2015). The emergence and spread of these carbapenemase-producing pathogens thus posed serious clinical treatment challenges and spurred renewed efforts to develop novel antimicrobial agents to treat such infections.

Ceftazidime-avibactam (CAZ-AVI) is an intravenously administered antimicrobial consisting of a third-generation cephalosporin ceftazidime and a novel non-β-lactam β-lactamase inhibitor avibactam, at a fixed ceftazidime: avibactam ratio of 4:1 (Shirley, 2018). It exhibited excellent *in vitro* activity against many extended-spectrum β-lactamase (ESBL)-, *Klebsiella pneumoniae* carbapenemase (KPC)-, AmpC-, and OXA-48-producing bacteria, including *Enterobacteriaceae* and *P. aeruginosa*, while showed no activity against metallo-β-lactamase (MBL)-producing strains or most *Acinetobacter* spp. isolates (Ehmann et al., 2012; Berkhout et al., 2015; Levasseur et al., 2015). CAZ-AVI has been demonstrated the clinical efficacy for the treatment of adults with complicated intra-abdominal infection (cIAI), complicated urinary tract infection (cUTI) [including pyelonephritis], hospital-acquired pneumonia (HAP) [including ventilator-associated pneumonia (VAP)], as well as other infections caused by aerobic Gram-negative organisms in patients with limited treatment options, ever since been approved by US Food and Drug Administration (FDA) in 2015 (Shirley, 2018).

CAZ-AVI was generally well tolerated with most adverse events (AEs) being of mild to moderate intensity. Overall, the most commonly reported AEs (occurring in ≥ 5% of patients) across phase II and III clinical trials were positive direct Coombs test, nausea, and diarrhea. The reported frequency of acute kidney injury (AKI) ranged from 0.1% to 1% in its prescribing information. However, accumulating evidence suggests that the incidence of AKI in real-world experience is much higher (Shirley, 2018). Thus, we summarized and discussed the current evidence regarding on CAZ-AVI induced renal disorders and its dosage adjustment in patients with impaired renal function.

## 2 CAZ-AVI in phase II and III clinical trials

The clinical efficacy and safety of ceftazidime-avibactam in the treatment of cUTI, cIAI, and HAP (including VAP) was demonstrated in phase II and III trials with carbapenem comparators. Herein, we summarized CAZ-AVI related renal impairment in clinical trials.

### 2.1 cUTI

In 2012, Vazquez et al. conducted a phase II study (NCT00690378) to compare the efficacy and safety of CAZ-AVI and imipenem-cilastatin in hospitalized adults with serious cUTI (Vazquez et al., 2012). Over the course of the study, serious adverse events (SAEs) were reported in 6 (8.8%) patients in the CAZ-AVI arm and 2 (3.0%) patients in the imipenem-cilastatin arm, from which acute renal failure was observed in 1 (1.5%) patient in the CAZ-AVI arm, and none was observed in the imipenem-cilastatin arm (Table 1, Entry 1). Bradley et al. evaluated the efficacy and safety of CAZ-AVI in children (≥ 3 months to < 18 years) with cUTI through a phase II study (NCT02497781) in 2019 (Bradley et al., 2019b). Similarly, nephrolithiasis was observed in 1 (1.5%) patient in the CAZ-AVI arm and none was observed in the cefepime arm (Table 1, Entry 2). However, in the phase III RECAPTURE program (Wagenlehner et al., 2016) and REPRISE trial (Carmeli et al., 2016) that compared the efficacy and safety of CAZ-AVI and carbapenems in patients with cUTI (REPRISE trial also analyzed the patients with cIAI), no renal impairment was reported (Table 1, Entries 3–4).

**TABLE 1:** Summary of CAZ-AVI associated renal impairment in phase II and III clinical trials.

Entry	Study ID	Type of infection	Design of study (No.)	CAZ-AVI (No.) Vs. Comparator (No.)	CAZ-AVI n (%)			Comparator n (%)		
					AEs	SAEs	Renal impairment	AEs	SAEs	Renal impairment
1	Vazquez et al. (2012)	cUTI	Phase II trial (135)	CAZ-AVI (68) Vs. Imipenem-cilastatin (67)	46 (67.6%)	6 (8.8%)	Renal failure acute: 1 (1.5%)	51 (76.1%)	2 (3.0%)	0
2	Bradley et al. (2019)	cUTI	Phase II trial (95)	CAZ-AVI (67) Vs. Cefepime (28)	36 (53.7%)	8 (11.9%)	Nephrolithiasis: 1 (1.5%)	15 (53.6%)	2 (7.1%)	0
3	Wagenlehner et al. (2016)	cUTI	Phase III trial RECAPTURE (1033)	CAZ-AVI (511) Vs. Doripenem (509)	185 (36.2%)	21 (4.1%)	Not mentioned	158 (31.0%)	12 (2.4%)	Not mentioned
4	Carmeli et al. (2016)	cUTI + cIAI	Phase III trial REPEISE (333)	CAZ-AVI (165) Vs. Mostly carbapenem (168)	51 (31%)	9 (5.5%)	Not mentioned	66 (39%)	10 (5.9%)	Not mentioned
5	Lucasti et al. (2013)	cIAI	Phase II trial (204)	CAZ-AVI with metronidazole (101) Vs. Meropenem (102)	65 (64.4%)	9 (8.9%)	Not mentioned	59 (57.8%)	11 (10.8%)	Not mentioned
6	Bradley et al. (2019)	cIAI	Phase II trial (83)	CAZ-AVI with metronidazole (61) Vs. Meropenem (22)	32 (52.5%)	5 (8.2%)	Renal colic: 1 (1.6%)	13 (59.1%)	1 (4.5%)	0
7	Mazuski et al. (2016)	cIAI	Phase III program RECLAIM (1066)	CAZ-AVI with metronidazole (529) Vs. Meropenem (529)	243 (45.9%)	42 (7.9%)	Renal disorder: 12 (2.3%)	227 (42.9%)	40 (7.6%)	Renal disorder: 3 (0.9%)
8	Qin et al. (2017)	cIAI	Phase III study RECLAIM in Asia subset (432)	CAZ-AVI with metronidazole (215) Vs. Meropenem (217)	82 (38.1%)	9 (4.2%)	Renal disorder: 1 (0.5%)	83 (38.2%)	11 (5.1%)	Renal disorder: 1 (0.5%)
9	Rodgers et al. (2022)	cIAI	Phase III study RECLAIM in Indian subset (125)	CAZ-AVI with metronidazole (62) Vs. Meropenem (63)	44 (71%)	1 (1.6%)	Dysuria: 1 (1.6%); Urinary retention: 5 (8.1%)	35 (55.6%)	1 (1.6%)	Dysuria: 3 (4.8%); urinary retention:3 (4.8%)
10	Torres et al. (2018)	HAP	Phase III trial REPROVE (879)	CAZ-AVI (405) Vs. Meropenem (403)	302 (75%)	75 (19%)	Not mentioned	299 (74%)	54 (13%)	Not mentioned

### 2.2 cIAI

In 2013, Lucasti et al. conducted a phase II trial (NCT 00752219) aimed to evaluate the safety and efficacy the CAZ-AVI plus metronidazole compared with meropenem in hospitalized patients with cIAI (Lucasti et al., 2013). The incidence of treatment-emergent AEs was similar to each other, and no renal failure was observed (Table 1, Entry 5). In another phase II study (NCT02475733), Bradley et al. also evaluated the safety and efficacy of CAZ-AVI plus metronidazole compared with meropenem in children with cIAI (Bradley et al., 2019), and renal colic occurred in 1 (1.6%) patient in CAZ-AVI plus metronidazole group (Table 1, Entry 6). In 2016, Mazuski et al. conducted a multicenter global phase III RECLAIM program that enrolled 1066 patients with cIAI and proved that CAZ-AVI plus metronidazole was non-inferior to meropenem across all populations (Mazuski et al., 2016). AEs occurred at a similar frequency in the two treatment groups, and renal disorders were observed in 12 (2.3%) and 3 (0.9%) patients in the CAZ-AVI plus metronidazole and meropenem arms, respectively (Table 1, Entry 7). Qin et al. and Rodgers et al. reinvestigated the safety and efficacy results of the Asian and Indian population subset from the RECLAIM trial (Qin et al., 2017; Rodgers et al., 2022). In the Asian subset, renal disorder was observed in 1 (0.5%) patient in both groups, while in the Indian subset, dysuria and urinary retention were newly reported (Table 1, Entries 8–9).

### 2.3 HAP

In 2018, Torres et al. conducted a phase III REPROVE trial to assess the efficacy and safety of CAZ-AVI in patients with nosocomial pneumonia, compared with meropenem (Torres et al., 2018). SAEs occurred in 75 (19%) patients in the CAZ-AVI arm and 54 (13%) patients in the meropenem arm, while renal disorders were not mentioned in both groups (Table 1, Entry 10).

In a word, renal impairment was rare in patients treated with CAZ-AVI in phase II and III clinical trials, although slightly higher than in the carbapenems group.

## 3 CAZ-AVI in real-world studies

As mentioned above, CAZ-AVI showed potent activity against class A (including ESBLs and KPC-type carbapenemases), class C (AmpC), and some class D (including OXA-48) β-lactamase-producing bacteria, mainly belong to CRE and CRPA. Herein, we summarized the safety data related to renal impairment reported in real-world for MDR Gram-negative infections (MDR-GNIs), as well as the safety data in the critically ill patients. We also compared the data about the incidence of AKI among patients receiving CAZ-AVI or polymyxins in the treatment of MDR-GNIs.

### 3.1 CAZ-AVI in the treatment of MDR-GNIs

In 2019, Jorgensen et al. conducted a multicenter, retrospective cohort study that enrolled 203 patients treated with CAZ-AVI for MDR-GNIs, from which CRE and CRPA were isolated from 117 (57.6%) and 63 (31.0%) culture specimens, respectively (Jorgensen et al., 2019). With regards to safety, 10 (4.9%) patients developed AKI, and 9 of them were receiving concomitant nephrotoxic agents (especially aminoglycosides or polymyxins) around the time of the event (Table 2, Entry 1).

**TABLE 2:** Summary of CAZ-AVI associated renal impairment in real-world studies.

Entry	Study ID	Type of infection (main pathogens)	Design of study	Patients included (No.)	CAZ-AVI n (%)	Comparator n (%)
					Renal impairment	Renal impairment
1	Jorgensen et al. (2019)	CRE and CRPA	Retrospective study	CAZ-AVI (203)	AKI: 10 (4.9%)	
2	Shields et al. (2016)	CRE	Retrospective study	CAZ-AVI (37)	AKI: 3 (10%)	
3	King et al. (2017)	CRE	Retrospective study	CAZ-AVI (60)	CRRT: 14 (23%)	
4	Sousa et al. (2018)	CRE (OXA-48)	Prospective study	CAZ-AVI (57)	AKI: 2 (3.5%)	
5	Da la Calle et al. (2019)	CRE (OXA-48)	Retrospective study	CAZ-AVI (24)	AKI: 2 (8.3%)	
6	Falcone et al. (2021)	MBL–producing Enterobacterales	Prospective study	CAZ-AVI plus ATM (54) Vs. Others (50)	AKI: 1 (1.9%)	AKI:10 (20%)
7	Caston et al. (2017)	CRE in hematologic cancer	Retrospective study	CAZ-AVI (8) Vs. Others (23)	Renal failure: 2 (25%)	Renal failure: 7 (30%)
8	Tsocaki et al. (2020)	CRE (KPC) in critically ill, mechanically ventilated patients	Retrospective study	CAZ-AVI (41) Vs. Others (36)	CRRT: 2 (5%)	CRRT: 4 (11%)
9	Chen et al. (2021)	CRE in Liver transplantation recipients	Retrospective study	CAZ-AVI (21)	AKI: 3 (14.3%)	
10	Feldman et al. (2022)	CRKP in patients with liver cirrhosis	Retrospective study	CAZ-AVI (15) Vs. Others (24)	AKI: 5 (33%)	AKI: 9 (38%)
11	Shields et al. (2017)	CRE (KPC)	Retrospective study	CAZ-AVI (13) Vs. Others (96)	AKI: 2 (18%)	AKI: CB + COL: 13 (57%); CB + AG:8 (44%); Others: 6 (18%)
12	Van Duin et al. (2018)	CRE	Prospective study	CAZ-AVI (38) Vs. COL (99)	Incident renal failure: 1 (4%)	Incident renal failure: 6 (13%)
13	Hakeam et al. (2021)	CRE	Retrospective study	CAZ-AVI (32) Vs. COL (29)	AKI: 3(9.4%)	AKI: 3 (10.3%)
14	Almangour et al. (2022)	CRE (KPC)	Retrospective study	CAZ-AVI (149) Vs. COL (81)	AKI: 23(15%)	AKI: 27 (33%)
15	Zheng et al. (2022)	CRE (CRKP)	Retrospective study	CAZ-AVI (82) Vs. Polymyxin B (82)	AKI: 0	AKI: 7 (8.5%)
16	Satlin et al. (2022)	CRE	Retrospective study	CAZ-AVI (21) Vs. Polymyxins (26)	AKI: 5 (22%)	AKI: 7 (26%)
17	Doremus et al. (2021)	MDR-GNIs	Retrospective study	BLBLIs (256) Vs. COL (256)	Overall AKI: 34 (13.3%); AKI without baseline renal disease: 14 (6.8%)	Overall AKI: 61(23.8%); AKI without baseline renal disease:36 (17.1%)

For the treatment of patients with CRE infection, Shields et al. conducted a retrospective study enrolled 37 patients in 2016 (Shields et al., 2016), and mentioned that 3 (10%) patients developed AKI, including 1 patient in combination with colistin (COL) (Table 2, Entry 2). In 2017, King et al. also described a retrospective review of 60 patients with CRE infection, from which 33 (55%) patients required renal dose adjustment, and 14 (23.3%) of these patients underwent further continuous renal replacement therapy (CRRT) (King et al., 2017) (Table 2, Entry 3).

Although KPC-type carbapenemase-producing *Enterobacterales* (CPE) were the most frequently isolated organisms, the experience in the treatment for OXA-48 CPE has also been reported. Sousa et al. designed a prospective observational study that enrolled 57 patients receiving CAZ-AVI for any infection produced by OXA-48 CPE (Sousa et al., 2018), and 2 (3.5%) patients developed AKI with one of them on concomitant COL during the treatment (Table 2, Entry 4). Da la Calle et al. also reviewed the characteristics of OXA-48 CPE infection (De la Calle et al., 2019), and 2 (8.3%) patients showed impaired renal function with neurological symptoms (Table 2, Entry 5). On the other hand, CAZ-AVI plus aztreonam (ATM) has also been considered as a potential therapeutic option for metallo-β-lactamase (MBL)-producing *Enterobacterales*. Falcone et al. conducted a prospective study enrolled 102 patients with bloodstream infections (BSI) due to MBL-producing *Enterobacterales* (Falcone M et al., 2021), and 1 (1.9%) patient developed drug-induced AKI (Table 2, Entry 6).

### 3.2 CAZ-AVI usage of critically ill patients

Critically ill patients, characterized by immune suppression, are a particularly vulnerable subpopulation to MDR-GNIs during hospitalization and after hospital discharge. Thus, the safety data of CAZ-AVI in these patients were also reviewed. Castón et al. (2017) conducted a multicenter retrospective study that included 31 patients with hematologic malignancies, from which 8 patients received CAZ-AVI treatment and two of them (25%) developed renal failure during treatment (Table 2, Entry 7). In 2020, Tsolaki et al. conducted a retrospective observational cohort study to evaluate the effectiveness of CAZ-AVI in critically ill, mechanically ventilated patients (Tsolaki et al., 2020), and 2 (5%) patients in the CAZ-AVI group required initiation of CRRT (Table 2, Entry 8). Liver transplantation (LT) recipients with carbapenem-resistant *Klebsiella pneumoniae* (CRKP) infection who received CAZ-AVI treatment were also reviewed retrospectively, and AEs were assessed by Haomin Zhang group (Chen et al., 2021). Three (14.3%) patients developed AKI, and two of them need further hemodialysis (Table 2, Entry 9). Recently, Feldman et al. also conducted a retrospective analysis that enrolled 39 patients with liver cirrhosis and CRKP infection (Feldman et al., 2022), and 5 (33%) patients in the CAZ-AVI group developed AKI (Table 2, Entry 10).

### 3.3 CAZ-AVI versus polymyxins in the treatment of MDR-GNIs

With the increasing prevalence of antibiotic resistance, polymyxins and CAZ-AVI have been used as the last-line therapeutic option for the treatment of MDR-GNIs, thus a comparison of safety, particularly in renal disorders, between CAZ-AVI and polymyxins was made. In 2017, Shields et al. conducted a retrospective study to compare the outcomes of patients treated with CAZ-AVI versus comparators for CRE infections (Shields et al., 2017). At the end of treatment, AKI rates were 18% (2/11), 57% (13/23), 44% (8/18), and 18% (6/33) for CAZ-AVI, carbapenem (CB) + COL, CB + aminoglycoside (AG), and other regimens, respectively (Table 2, Entry 11). AKI incidence was significantly higher among patients receiving AG or COL, and it was significantly more common with COL-containing than with AG-containing regimens.

In 2018, Van Duin et al. conducted a prospective multicenter study that enrolled 38 patients treated with CAZ-AVI and 99 with COL in the treatment of infections due to CRE (Van Duin et al., 2018). The inverse probability of treatment weighting (IPTW)-adjusted estimates for not observed die with incident renal failure was 4% (1/38) and 13% (6/99) for CAZ-AVI and COL groups, respectively (Table 2, Entry 12). Hakeam et al. conducted a retrospective, multicenter study that included 61 patients with CRE treated with CAZ-AVI or COL (Hakeam et al., 2021), and no difference in AKI development between CAZ-AVI and COL groups was observed (9.4% and 10.3%, respectively) (Table 2, Entry 13). Almangour et al. also compared the safety and effectiveness of CAZ-AVI to COL-based regimen in the treatment of infections caused by CRE (Almangour et al., 2022), while AKI was significantly less common in patients who received CAZ-AVI than COL (15% and 33%, respectively) (Table 2, Entry 14).

Recently, Zhang et al. conducted a retrospective study in two Chinese tertiary hospitals for critically ill patients with CRKP infection who received CAZ-AVI or polymyxin B (PMB)-based treatment (Zheng et al., 2022). According to safety evaluation results, 7 (8.5%) patients developed AKI in the PMB-based group, while AKI was not mentioned in CAZ-AVI-based patients (Table 2, Entry 15). In a study that assessed the impact of a rapid molecular test for KPC, the outcomes of CAZ-AVI and polymyxin (including COL and PMB) targeted therapies were also evaluated (Satlin et al., 2022). 5 (22%) and 7 (26%) patients developed AKI in CAZ-AVI and polymyxin groups, respectively (Table 2, Entry 16). In 2021, Doremus et al. evaluated the incidence of AKI among patients receiving COL or novel β-lactam β-lactamase inhibitors (BLBLIs). The overall AKI incidence was 13.3% and 23.8% in BLBLIs and COL groups, respectively (Doremus et al., 2021). For patients without baseline renal disease, the odds of AKI in patients on COL were three times higher than that of patients receiving BLBLIs agents (17.1% vs. 6.8%) (Table 2, Entry 17).

In short, CAZ-AVI associated AKI was more frequent in real-world than in phase II and III clinical trials. Although the underlying mechanism of CAZ-AVI associated AKI is unclear, it is likely that concomitant nephrotoxin exposure and special disease status may play an essential role.

## 4 Pharmacokinetic/pharmacodynamic targets and dosage adjustment of CAZ-AVI

In 2015, both Das et al. and Merdjan et al. assessed the pharmacokinetic (PK) and safety profiles of CAZ-AVI and whether drug-drug interactions existed between each other (Das et al., 2015; Merdjan et al., 2015). The results indicated that both ceftazidime and avibactam exhibited approximate dose linearity when administered in combination with clinically relevant doses, and the PK of avibactam is unaffected when administered alone or with ceftazidime. On the other hand, neither ceftazidime nor avibactam appear to undergo significant metabolism, and both drugs are primarily eliminated unchanged in the urine (Shirley, 2018). Vishwanathan et al. also evaluated the metabolism and drug-drug interaction potential of ceftazidime and avibactam. They suggested that ceftazidime mainly excreted through glomerular filtration, while the elimination of avibactam involved active tubular secretion in addition to glomerular filtration (Vishwanathan et al., 2014). Additionally, CAZ-AVI shares the typical PK features of β-lactams (BLs), such as hydrophilicity, low plasma protein binding, low molecular weight, and small volume of distribution (V_D_) (Yahav et al., 2020). Consistently, dosage adjustments are recommended in patients with impaired renal function (Giri et al., 2019).

On the other hand, ceftazidime exhibits time-dependent pharmacodynamics (PD), and its effect is related to the percentage of time that free drug concentration remains above the minimum inhibitory concentration (MIC) of the targeted pathogen (% fT > MIC) (Sader et al., 2017; Nichols et al., 2018a). For avibactam (in combination with ceftazidime), the PK/PD index was defined as the free time above a critical concentration(C_T_)below which sufficient inhibition of ceftazidime was lost (% fT > C_T_) (Coleman et al., 2014; Berkhout et al., 2016; Nichols et al., 2018b). Ceftazidime and avibactam were coadministered in a fixed dose ratio (4:1), and a joint probability of target attainment (PTA) was calculated to guide the dosage regimen selection and validation on the basis of the simultaneous achievement of separate PK/PD targets and population PK modeling through Monte Carlo simulation (Li et al., 2018; Gatti et al., 2024). According to the nonclinical studies, the joint PTA of CAZ-AVI was defined as ceftazidime 50% fT > 8 mg/L and avibactam 50% fT > 1 mg/L, and dosage selection was dependent on the achievement of a high (>90%) joint PTA (Li et al., 2018; Das et al., 2019; Li et al., 2020). In a phase III clinical trial, the joint PTA analyses supported the CAZ-AVI dosage regimen of 2.5 g q8 h for patients with estimated creatinine clearance (CrCl) above 50 mL/min, and modified dosage adjustment for patients with moderate or severe renal impairment (CrCl < 50 mL/min) (Li et al., 2020).

In consideration of the fact that the degree of renal dysfunction is an essential factor in dosage adjustment for patients with renal impairment, we are wondering how to conduct renal dosage adjustment to achieve equivalent exposures in patients with AKI. Indeed, a phase I study demonstrated that increased severity of renal impairment was associated with decreased total plasma clearance (CL) of avibactam, as previously observed for ceftazidime (Nicolau et al., 2015). However, a Phase III clinical study suggested that in patients with moderate renal dysfunction, the efficacy of CAZ-AVI was worse than that of meropenem, which was related to insufficient dosage of CAZ-AVI (Mazuski et al., 2016). In this trial, patients with normal kidney function receiving 2.5 g q8 h CAZ-AVI showed a higher response rate compared to patients with moderate renal impairment treated with 1.25 g q12 h. Moreover, in patients with moderate renal impairment, there was a lower response rate in the CAZ-AVI arm (1.25 g q12 h) in comparison with the meropenem arm (1 g q12 h; 45.2% vs. 74.3%; *p* = 0.016), which potentially was a result of a higher proportional dose reduction in CAZ-AVI arm than in meropenem arm (66% vs. 33%). Notably, among patients with moderate renal impairment at baseline, 67.9% showed improvements to CrCl above 50 mL/min within 48–72 h (Mazuski et al., 2016). Consequently, the recommended dose of CAZ-AVI in patients with moderate renal impairment was increased from 1.25 g q12 h to 1.25 g q8 h based on the above-mentioned PK/PD targets to achieve a higher joint PTA (Li et al., 2020). Likewise, the modified dosage adjustment was validated in another phase III trial (Torres et al., 2018) and thus applied in real-world experience (Jorgensen et al., 2019). In 2019, Crass et al. (2019) retrospectively reviewed the records of 18,500 patients with infectious diseases, and they identified that the overall rate of AKI on admission was 17.5%, with 57.2% of cases achieving kidney injury resolution by 48 h. Besides that, 47.9% of patients with moderate renal impairment on admission had an improvement of CrCl above 50 mL/min within 48 h (Crass et al., 2019), which was consistent with the results of the above phase III study (Mazuski et al., 2016). These data highlight the dynamic nature of renal function and the potential for rapid recovery in patients with AKI. Therefore, unnecessary dose reduction in the setting of transient AKI may have played a role in the decreased clinical response in patients with moderate renal impairment.

In fact, AKI is a dynamic perturbation of renal steady-state, which makes it challenging to conduct an accurate characterization of patient kidney function. The current renal dose adjustment protocols are based on small, early-phase PK studies that enroll patients with stable chronic kidney disease (CKD) before testing in registered clinical trials. Of note, the most common formulas to evaluate kidney function are Cockcroft-Gault, Modification of Diet in Renal Disease (MDRD), and CKD epidemiology collaboration (CKD-EPI). However, these equations are based on the serum creatine (Scr) under the steady-state conditions and may be inaccurate to estimate renal function in the dynamic setting of AKI. Therefore, these paradigmas are appropriate for maintenance therapeutics in CKD patients, and may overestimate dose reductions for patients with AKI in ultimately clinical practice (Crass et al., 2019). Moreover, the kinetic eGFR equation has also been developed to assess eGFR in the setting of dynamic renal states, which included the magnitude to which Scr concentration was increased or decreased relative to the baseline steady-state value and the rapidity of the variation tendency. Rather than relying on a single Scr concentration, this equation was based on multiple Scr measurements and the mathematics of creatinine mass balance (Chen, 2013). However, it does not take into consideration the creatinine production due to infection and loss of muscle mass, and changes in V_D_ in acutely ill patients (Chen, 2018), as significant alterations of V_D_ may occur in patients with AKI, thus affecting the drug exposure profile (Bidell and Lodise, 2018). As a consequence, the inappropriate empirical dose reduction in the setting of transient AKI may contribute to the decreased clinical response in patients with moderate renal impairment. On the other hand, it has been well documented that adequate antibiotic therapy within the first 48 h is a significant determinant of outcomes for critically ill patients (Leibovici et al., 1998; Lee et al., 2017; Crass et al., 2019). Patients with severe infections often show dynamic changes in CrCl, and prompt recovery of renal function generally occurs within the first 48 h as a result of fluid resuscitation or other supportive care (Gatti and Pea, 2021). Thus, to minimize the risk-to-benefit ratio, standard dosing of CAZ-AVI (2.5 g q8 h) may be optimal for patients with and without renal dysfunction during the first 48 h. Deferral of renal dosage adjustment should be applied only after 48 h for patients with persistent AKI (Lewis and Muller, 2016; Crass et al., 2019; Gorham et al., 2022).

Except for the frequently occurred AKI mentioned in this study, other renal variations are also observed in critically ill patients, like renal replacement therapy (RRT) or augmented renal clearance (ARC), thus data about appropriate dosage regimens based on PK/PD targets are strongly needed under these circumstances (Gatti and Pea, 2021; Gorham et al., 2022). It has been demonstrated that 5%–10% of critically ill patients with AKI eventually require RRT during their hospital stay (Tolwani, 2012). Wenzler et al. (2017) found that a 1.25 g q8 h CAZ-AVI could achieve optimal drug concentrations (100% fT > MIC 6 mg/L) against MDR-GNIs in critically ill patients on continuous venovenous hemofiltration (CVVH). Conversely, in a case report of a 50-year-old critically ill patient with MDR *Pseudomonas aeruginosa* (MIC 8 mg/L) pneumonia receiving continuous venovenous hemodiafiltration (CVVHDF), only standard drug regimens (2.5 g q8h) could achieve high trough concentrations (Soukup et al., 2019). Moreover, in a study of 77 patients treated with CAZ-AVI for CRE infections, high emergence of resistance was reported in patients requiring RRT, which was defined as an independent predictor of clinical failure and development of resistance (Shields et al., 2018). As such, in critically ill patients receiving RRT, a drug regimen of 1.25 g q8 h could be used for susceptible strains (MIC < 4 mg/L), while higher regimens and/or prolonged infusion, even continuous infusion, should be considered for less susceptible strains (Fresan et al., 2023).

A recent study indicated that ARC may cause higher drug clearance and underexposure, thus resulting in poor clinical outcomes (Cook and Hatton-Kolpek, 2019). A subgroup analysis of 239 patients with ARC included in the REPROVE trial showed that the standard dosage of 2.5 g q8 h over 2 h ensured > 95% PTA of 50% fT > MIC up to 16 mg/L, despite a 35% decrease in drug exposure compared to patients with normal renal function (Torres et al., 2018). In 2019, Stein et al. conducted a PK/PD analysis of CAZ-AVI in 10 critically ill patients, of whom two had ARC. Serum concentrations of ceftazidime and avibactam were measured individually, and optimal joint PTA was achieved under the current dosage regimens of CAZ-AVI, including these two with ARC treated with 2.5 g q8 h over 2 h (Stein et al., 2019). In a study evaluating the efficacy of different CAZ-AVI dosage regimens against some *Enterobacteriaceae* members and *P. aeruginosa* by Monte Carlo simulation, patients with ARC failed to reach 90% cumulative fraction of response under the standard dose of 2.5 g q8 h (Dai et al., 2021). This phenomenon was also observed in a therapeutic drug monitoring (TDM) of CAZ-AVI concentration in CRKP-infected patients with different kidney statuses. In this study, two patients with ARC showed lower ceftazidime and avibactam serum concentrations, even though receiving 2.5 g q6 h CAZ-AVI (Teng et al., 2022). These results implied that increased dosing or dose optimization using prolonged duration may be needed in ARC patients to maintain therapeutic exposure.

As mentioned above, only conservative PK/PD targets of ceftazidime 50% fT > MIC 8 mg/L and avibactam 50% fT > C_T_ 1 mg/L were used in phase II/III clinical trials (Li et al., 2018; Das et al., 2019). However, several experiences in critically ill patients reported that higher PK/PD target achievement was related to better clinical outcomes. In 2014, Roberts et al. conducted a prospective study including 384 patients to define BLs levels in critically ill patients, and they found that positive clinical outcome was associated with more aggressive PK/PD targets of 100% fT > MIC compared to 50% fT > MIC (Roberts et al., 2014). Notably, a review describing PK/PD issues associated with renal dose adjustments of CAZ-AVI demonstrated that antibiotic exposure showed a close relationship not only with clinical outcomes but also with the emergence of resistance (Bidell and Lodise, 2018). Therefore, more aggressive PK/PD targets of ceftazidime 100% fT > 4–8 × MIC and avibactam 100% fT > C_T_ 4 mg/L were suggested to maximize bacteriological and clinical response, as well as suppress the emergence of resistance and prevent any toxicity risk (Gatti and Pea, 2021). This suggestion was consistent with the guidelines from the French Society of Pharmacology and Therapeutics (Société Française de Pharmacologie et Thérapeutique—SFPT) and the French Society of Anaesthesia and Intensive Care Medicine (Société Française d’Anesthésie et Réanimation—SFAR) for the optimization of BLs in critically ill patients (Guilhaumou et al., 2019). Furthermore, according to a recent definition, the joint PK/PD targets of CAZ-AVI were considered optimal when ceftazidime 100% fT > 4 × MIC and avibactam 100% fT > CT 4 mg/L (Gatti et al., 2023). Unfortunately, several risk factors, especially the variation in renal function, render the achievement of aggressive PK/PD targets unpredictable in critically ill patients. Hence, TDM-guided dosage adjustment may represent a helpful tool for achieving aggressive PK/PD targets, thus maximizing effectiveness and minimizing toxicity and resistance development (Gatti and Pea, 2021).

In sum, although alternative dosing strategies based on multiple daily dosing coupled with prolonged infusion may represent the best approach to maximize the time-dependent antimicrobial activity of BLs. However, it is worth noting that clinicians still face several challenges when making dosage optimization of CAZ-AVI, especially among critically ill renal patients. In all of the scenarios as mentioned above, implementation of adaptive real-time TDM focused on attaining more aggressive PK/PD targets of ceftazidime 100% fT > 4–8 × MIC and avibactam 100% fT > C_T_ 4 mg/L may be the most powerful strategy in maximizing the clinical response and in preventing the development of resistance.

## 5 Conclusion

CAZ-AVI was used for the treatment of cUTI, cIAI, HAP, and other infections caused by aerobic Gram-negative bacteria in patients with limited treatment options. It is an intravenously administered combination of ceftazidime and avibactam, which can be almost completely eliminated through glomerular filtration. In this review, we collected the safety data related to renal impairment from clinical trials and real-world studies. The results indicated that the incidence of AKI in real-world was significantly higher than reported in the local prescribing information. Therefore, CAZ-AVI should receive particular attention as a possible cause of renal disorders, especially for critically ill patients or concomitant administration with other nephrotic agents, such as polymyxin and aminoglycosides. Notably, AKI is transient in the majority of cases and may resolve within the first 48 h. Therefore, inappropriate dose reduction in this window may result in increased clinical failure. To minimize toxicity without compromising efficacy, we suggest an unadjusted dosing for 48 h with a subsequent renal dose reduction if renal impairment persists. Whether transient or persistent, renal impairment should be thoroughly evaluated so as to make informed decisions related to renal dosage adjustment. Besides, appropriate dosage adjustment should also be carefully taken into consideration in critically ill patients with ARC or requiring RRT. TDM-guided dosage optimization intended to achieve more aggressive PK/PD targets of ceftazidime 100% fT > 4–8 × MIC and avibactam 100% fT > C_T_ 4 mg/L may represent a helpful strategy in maximizing clinical response and minimizing toxicity and resistance development.
